# Causes of perinatal death at a tertiary care hospital in Northern Tanzania 2000–2010: a registry based study

**DOI:** 10.1186/1471-2393-12-139

**Published:** 2012-12-02

**Authors:** Blandina T Mmbaga, Rolv Terje Lie, Raimos Olomi, Michael Johnson Mahande, Oneko Olola, Anne Kjersti Daltveit

**Affiliations:** 1 Kilimanjaro Christian Medical Centre and Kilimanjaro Christian Medical College, P.O Box 3010, Moshi, Tanzania; 2 Department of Public Health and Primary Health Care, University of Bergen, P.O Box 7804, 5020, Bergen, Norway; 3 Centre for International Health, University of Bergen, P.O Box 7804, Bergen, Norway; 4 Norwegian Institute of Public Health, Division of Epidemiology, Oslo, Norway

**Keywords:** Perinatal mortality, Perinatal deaths, Maternal disease, Obstetric complication, NICE classification

## Abstract

**Background:**

Perinatal mortality reflects maternal health as well as antenatal, intrapartum and newborn care, and is an important health indicator. This study aimed at classifying causes of perinatal death in order to identify categories of potentially preventable deaths.

**Methods:**

We studied a total of 1958 stillbirths and early neonatal deaths above 500 g between July 2000 and October 2010 registered in the Medical Birth Registry and neonatal registry at Kilimanjaro Christian Medical Centre (KCMC) in Northern Tanzania. The deaths were classified according to the Neonatal and Intrauterine deaths Classification according to Etiology (NICE).

**Results:**

Overall perinatal mortality was 57.7/1000 (1958 out of 33 929), of which 1219 (35.9/1000) were stillbirths and 739 (21.8/1000) were early neonatal deaths. Major causes of perinatal mortality were unexplained asphyxia (n=425, 12.5/1000), obstetric complications (n=303, 8.9/1000), maternal disease (n=287, 8.5/1000), unexplained antepartum stillbirths after 37 weeks of gestation (n= 219, 6.5/1000), and unexplained antepartum stillbirths before 37 weeks of gestation (n=184, 5.4/1000). Obstructed/prolonged labour was the leading condition (251/303, 82.8%) among the obstetric complications. Preeclampsia/eclampsia was the leading cause (253/287, 88.2%) among the maternal conditions. When we excluded women who were referred for delivery at KCMC due to medical reasons (19.1% of all births and 36.0% of all deaths), perinatal mortality was reduced to 45.6/1000. This reduction was mainly due to fewer deaths from obstetric complications (from 8.9 to 2.1/1000) and maternal conditions (from 8.5 to 5.5/1000).

**Conclusion:**

The distribution of causes of death in this population suggests a great potential for prevention. Early identification of mothers at risk of pregnancy complications through antenatal care screening, teaching pregnant women to recognize signs of pregnancy complications, timely access to obstetric care, monitoring of labour for fetal distress, and proper newborn resuscitation may reduce some of the categories of deaths.

## Introduction

Perinatal mortality refers to the death of a fetus or death during first week of life, and is thought to reflect maternal pre-pregnancy health as well as maternal, obstetric, and newborn care. It is widely used as a health indicator in international comparisons, and within countries and regions to estimate temporal trends [1]. Globally, approximately 5.9 million perinatal deaths occur annually, of which 3.2 million stillbirths (SB) and 2.7 million early neonatal deaths (END) [2]. The highest burden of perinatal deaths is in developing countries which account for about 98% of all deaths [2].

Tanzania like other Sub Saharan African countries, has a high perinatal mortality estimated to be 69/1000 births in 2004 [2]. A recent national survey reported perinatal mortality for pregnancies lasting seven months or more to be 36/1000, ranging between 24/1000 and 60/1000 in the different zones [3]. In studies, estimates of perinatal mortality in Tanzania vary depending on the geographical area, the type of study, and information collected, ranging from 27–124 deaths/1000 births [4-10].

Causes and determinants of early neonatal deaths and stillbirths differ from causes of postneonatal deaths [1,11,12]. Many perinatal deaths are the consequence of a chain of events [13], in which complications such as obstructed labour and fetal malpresentation are common [1,12]. Globally, one third of all stillbirths (1.2 million) are estimated to occur during labour/delivery, while one third of all early neonatal deaths (0.9 million) are due to birth asphyxia [12]. These two causes of perinatal mortality represent intrapartum related perinatal deaths and are examples of deaths that are largely linked to quality of care around the time of delivery [1,2,12].

Several classification systems of perinatal deaths have been developed. The usefulness of these systems varies considerably due to dissimilarities in recording system and information collected [14]. The Wigglesworth classification [15] has also been used for classification of perinatal and neonatal deaths in developing countries because it is simple and does not require sophisticated investigations, aimed at subdividing cases into groups with clear implications for clinical management. The Neonatal and Intrauterine deaths Classification according to Etiology (NICE) is developed to uncover the underlying etiology which might have initiated the chain of events leading to death, in terms of maternal, obstetric, fetal and neonatal conditions [13,16]. Compared to Wigglesworth, the NICE classification is found to reduce the proportion of intrauterine deaths, deaths from asphyxia, and deaths from immaturity linked to maternal disease, abruption placenta or obstetric complications [13].

Due to lack of good vital registration systems, reports on perinatal and neonatal mortality in developing countries are mainly based on public health surveillance such as demographic and health surveys. Ninety percent of the children under the age of five in Tanzania are unregistered [17]. In high income countries where all births and deaths are registered, the information obtained has been continuously used for planning and implementation of prevention strategies. In low income countries, hospital records may be an available source of information, but these are usually difficult and time consuming to retrieve, and are not designed to fit into classification systems.

The Kilimanjaro Christian Medical Centre (KCMC) Medical Birth Registry system was established in 1999 as collaboration between Kilimanjaro Christian Medical College, Moshi, Tanzania and the University of Bergen, Norway through the support of the Norwegian Council for Higher education program for Development Research (NUFU) project. The birth registry at KCMC is a daily activity including public holidays with integrated neonatal registry for neonates admitted to a neonatal care unit. The KCMC Medical Birth Registry and neonatal registry include maternal, obstetric, fetal, and neonatal characteristics which give us the opportunity to classify perinatal deaths into etiologically based groups. The aim of this study was to identify and classify causes of perinatal deaths by using the Neonatal and Intrauterine death Classification according to Etiology (NICE), and possibly identify causal mechanisms relevant for prevention.

## Methods

### Setting

This study is based on data collected at KCMC hospital in Northern Tanzania. The hospital is a tertiary care and zonal referral hospital which serves about 10 million people from mainly four regions in Northern Tanzania, namely Kilimanjaro, Arusha, Tanga and Manyara. Being a tertiary referral hospital the KCMC labour ward receives normal deliveries as well as high risk mothers with maternal or obstetric complications referred at various stages of pregnancy or labour from Moshi urban area or from other health facilities in the Northern zone.

The KCMC obstetrics and gynaecology department has a team on call which includes one specialist or consultant obstetrician, one obstetric resident and one intern doctor, two anaesthesiologists and 3 midwives who take care of the department outside regular working hours for comprehensive emergency obstetrics and gynaecological care. The department has two operative theatres in labour ward for emergency caesarean sections. In the Kilimanjaro region 70% of all births take place at health facilities [18]. Around 50% of the deliveries at KCMC are from Moshi urban area. The caesarean section rate at the institution is about 33% [19].

**Figure 1:**
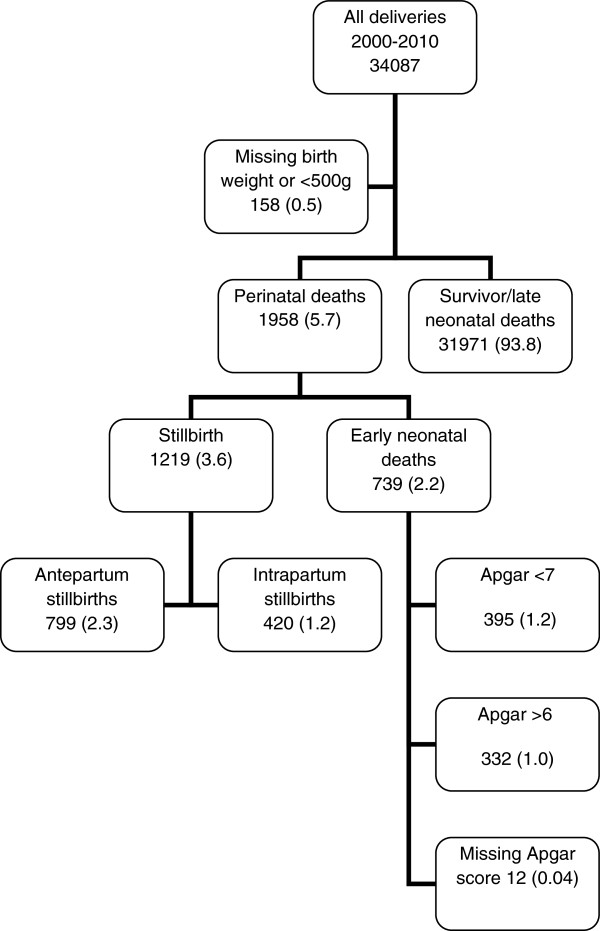
**Description of the study population.** Numbers in brackets are proportions of all births 2000–2010 (N=34087).

Based on records from the birth registry linked to the neonatal registry from July 2000 to October 2010 [20], we established a cohort of births with birth weight 500 g or more. A total of 34087 births were recorded of which 158 (0.4%) the birth weights were either missing or below 500 g (Figure 1). Therefore, our study population was 33929 births with birth weight 500 g or more, of which 1958 died perinatally.

### Data collection

Information on all mothers who delivered at KCMC was obtained through a structured questionnaire and the mothers being interviewed within the first 24 hours after delivery. Informed consent was obtained from mothers prior to the interview. Information was also extracted from the antenatal care record cards. Detailed description of the data collection procedure and data collected for the birth registry and neonatal registry have been previously published [20,21].

For stillbirths, time of death was recorded as before labour, during labour, or unknown. The status of the fetus was also recorded, whether it was a macerated stillbirth or fresh one. The information was also sought whether the fetus died before or after admission to labour ward. Reporting of early neonatal deaths included date of death, time of death (died within first 24 hours, died within first week), and up to three diagnoses of cause of death [20].

### Variable definitions

Early neonatal deaths include newborns that die during first week of life. We define perinatal mortality as stillbirth or early neonatal death with birth weight 500 grams or more [22]. Perinatal mortality rate (PNMR), stillbirth rate (SBR) and early neonatal mortality rate (ENMR), were calculated as follows: PNMR = (stillbirths + early neonatal deaths/total births) × 1000, SBR = (stillbirths/total births) × 1000 and ENMR = (early neonatal deaths/live births) × 1000.

Outcome was perinatal death, overall and according to cause of death. Causes of death were classified on the basis of maternal, obstetric, fetal and neonatal characteristics identified in the linked registry data, according to the NICE classification [23], with a mild modification of the *unexplained asphyxia* category (Table 1) based on our previous modification [20]. In a strictly hierarchical order, each stillborn or early neonatal death was classified into one of the 13 specific, mutually exclusive causes of death. For the two causes of death categories *maternal disease* and *obstetric complications*, we also investigated co-morbidity.

**Table 1:** Definitions of the characteristics included in the 13 categories of causes of perinatal deaths by NICE classification

**Causes**	Characteristics*
1. Congenital anomalies:	Include stillborn and liveborn infants with lethal malformations or potentially lethal malformations that markedly increase mortality risk.
2. Multiple births:	Includes multiple births other than duplex, or duplex in combination with immaturity (<33 gestational weeks) or intrauterine deaths.
3. Maternal disease:	Maternal diabetes mellitus if the infant is stillborn or is large for date (Z-score >2 SD). Maternal pre-eclampsia, renal disease, hepatosis, epilepsy, systemic lupus erythematosus (SLE) included when combined with an infant either small for date (Z-score <−2 SD) or immature (<33 gestational weeks), or dead before labour.
4. Specific fetal conditions:	Isoimmunization, unexplained hydrops featalis, tumors and specific fetal infections. Accidents included when combined with stillbirth.
5. Unexplained SGA infants:	Infants Z-score <−2.5 SD without any evidence of maternal disorder.
6. Placental abruption:	If combined with asphyxia, immaturity (<33 gestational weeks) or intrauterine death.
7. Obstetric complications:	Uterine rupture, disproportion, malpresentation, cord prolapse, cord compression, placenta previa, foetal blood loss and precipitated labour.
8. Unexplained antepartum stillbirths	<37 gestational weeks
9. Unexplained antepartum stillbirths	>36 gestational weeks
10. Specific infant conditions:	Liveborn infants >32 gestational weeks with septicaemia, meningitis or pnaeumonia, includes term infants with respiratory distress syndrome (RDS) or sudden infant death syndrome (SIDS). Accidents included when causing neonatal death.
11. Unexplained asphyxia:	Intrapartum death, deaths occur < 4 hrs after birth and cases with **Apgar score <7 at 5 min**, where the asphyxia is not explained, **clinical diagnosis Hypoxic ischaemic encephalopathy (HIE) or severe birth asphyxia where Apgar score is missing** and the case does not belong to groups 1–10 above. Immature infants 27 gestational weeks or **< 1000 g** are excluded.
12. Unexplained immaturity:	Liveborn infants <33 gestational weeks and 2500 g (or 1800 g if gestational age is unknown) where the immaturity is not explained and the case does not belong to groups 1–11 above.
13. Unclassifiable cases:	Cases not in groups 1–12.

*Characteristics included in the 13 categories adapted and modified from Winbo et al. [23]. Modifications are bolded.

Main results were stratified according to referral status (mother referred for delivery due to medical condition yes/no). The following conditions recorded in the birth registry were considered; obstructed labour, malpresentation, prolonged labour, retained twin, fetal distress, cord prolapse, premature/prolonged rupture of membrane, abruption placenta, placenta previa, antepartum haemorrhage, ruptured uterus, preeclampsia, eclampsia, gestational or diabetic mellitus, hypertension, and malaria. Referral due to previous caesarean section without any of the complications above was not regarded a medical referral.

### Data analysis

Data were analyzed using Statistical Package for Social Science (SPSS) program for Windows Version 19.0 (SPSS 19.0 Chicago Inc. III, USA). Descriptive measures such as mean, standard deviation, rate per 1000 and relative risk were calculated.

### Ethical approval

The protocol for this study was approved by Kilimanjaro Christian Medical college (KCM-College) research ethics committee, with certificate no. 333 of 15^th^ July 2010.

## Results

Among the 1958 perinatal deaths 1026 (52.4%) were males, 917 (46.8%) were females, 15 (0.8%) had unknown sex, 1017 (51.9%) were below 2500 g, and 781 (39.9%) were born before 37 weeks of gestation. Mean (SD) birth weight and gestational age were 2335 (944) g and 36 (4.7) weeks, respectively. Mean (SD) maternal age and number of ANC visits were 28.2 (6.4) years and 3.8 (2.0), respectively. Gestational age was missing in 247 (12.6%) perinatal deaths (151 stillbirths and 96 early neonatal deaths), and Apgar score at 5 minutes was missing in 12 (1.6%) early neonatal deaths. Mode of delivery for the perinatal deaths was spontaneous vaginal delivery (55.6%), cesarean section (35.3%), assisted breech delivery (5.6%), vacuum extraction (1.7%), destructive operative delivery (0.2%), and unknown (1.5%). Corresponding numbers for all births were 64.5%, 32.8%, 1.2%, 1%, 0.01% and 0.4%. In addition, 0.02% of all births were delivered by forceps.

**Figure 2:**
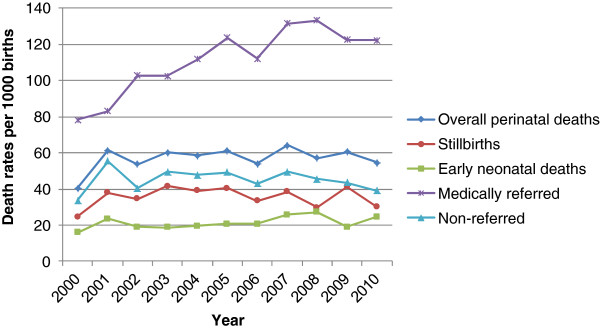
**Trends in stillbirths,early neonatal deaths and perinatal deaths at KCMC 2000–2010**.

Overall perinatal mortality was 57.7/1000 births (1958 out of 33 929) (Table 2), of which 1219 (35.9/1000) were stillbirths and 739 (21.8/1000) were early neonatal deaths. The majority of the stillbirths (799, 65.5%) were antepartum stillbirths, while 420 (34.5%) were intrapartum stillbirths. Overall and among non-referred, there were no time trends in perinatal mortality from 2000 to 2010 (Figure 2). In the referred group, mortality increased from around 80/1000 to more than 120/1000.

**Table 2:** **Number and rate of stillbirths and early neonatal deaths by birth weight among 1958 perinatal deaths at KCMC 2000**-**2010**

		**Birth weight in grams**		
	Total	<1500	1500-2499	>=2500
	n (/1000)	n (/1000)	n (/1000)	n (/1000)
***Total births***	**33929**	**741**	**3787**	**29401**
***Perinatal deaths***	***1958*** (***57***.***7***)	***459*** (***619***.***4***)	***558*** (***147***.***3***)	***941*** (***32***.***0***)
Stillbirths	1219 (35.9)	283 (381.9)	383 (101.1)	553 (18.8)
Antepartum (Macerated) stillbirths	799	204	272	323
Intrapartum (Fresh) stillbirths	420	79	111	230
Early neonatal deaths	739 (21.8)	176 (237.5)	175 (46.2)	388 (13.2)
Apgar <7	395	97	73	215
Apgar ≥7	332	77	98	167
Missing Apgar score	12	2	4	6
***Total singletons***	**32165**	**552**	**2916**	**28697**
Perinatal deaths	1752 (54.5)	348 (630.4)	491 (168.4)	913 (31.8)
***Total multiple births***	**1765**	**190**	**871**	**704**
Perinatal deaths	206 (116.7)	111 (584.2)	67 (76.9)	28 (39.8)

### Causes of perinatal death

Overall, major causes of perinatal death were *unexplained asphyxia* (n=425, 12.5/1000), *obstetric complications* (n=303, 8.9/1000), *maternal disease* (n=287, 8.5/1000), *unexplained antepartum stillbirths after 37 weeks of gestation* (n= 219, 6.5/1000), and *unexplained antepartum stillbirths before 37 weeks of gestation* (n=184, 5.4/1000), (Table 3). In the large group of *unexplained asphyxia*, 236 (55.5%) were early neonatal deaths and 189 (44.5%) were intrapartum stillbirths. A further analysis of co-morbidities showed that obstructed/prolonged labour was present in more than 80% of the deaths in the *obstetric complications* category, and that preeclampsia/eclampsia was present in nearly 90% of the deaths in the *maternal disease* category.

**Table 3:** **Causes of stillbirths and early neonatal deaths by Neonatal and Intrauterine Classification of death according to Etiology** (**NICE**) (**N**=**1958**, **birth weight** ≥**500 g**)

		**Medical referral**		
	**All**	**No**	**Yes**	**RR**
Number of births	33929	27460	6469	
Identified causes	n (/1000)	n (/1000)	n (/ 1000)	
1. Congenital anomalies	84 (2.5)	53 (1.9)	31 (4.8)	2.5
2. Multiple birth	156 (4.6)	110 (4.0)	46 (7.1)	1.8
3. Maternal disease	287 (8.5)	150 (5.5)	137 (21.8)	4.0
4. Specific fetal conditions	1 (0.03)	1 (0.04)	0	0
5. Growth restriction	70 (2.1)	52 (1.9)	18 (2.8)	1.5
6. Placental abruption	75 (2.1)	23 (0.8)	52 (8.0)	10.0
7. Obstetric complications	303 (8.9)	57 (2.1)	246 (38.0)	18.1
8. Unexplained antepartum stillbirth <37 weeks	184 (5.4)	144 (5.2)	40 (6.2)	1.2
9. Unexplained antepartum stillbirth ≥37 weeks	219 (6.5)	173 (6.3)	46 (7.1)	1.1
10. Specific infant conditions	33 (1.0)	29 (1.1)	4 (0.6)	0.6
11. Unexplained asphyxia	425 (12.5)	353 (12.9)	72 (11.1)	0.9
12. Unexplained immaturity	46 (1.4)	39 (1.4)	7 (1.1)	0.8
13. Unexplained	75 (2.1)	69 (2.5)	6 (0.9)	0.4
Total	1958 (57.7)	1253 (45.6)	705 (109)	2.4

RR =PMR medical referral/PMR non referral.

Births to mothers referred for delivery due to medical conditions accounted for 19.1% of all births and 36% of all deaths. Perinatal mortality was 45.6 per 1000 in the non-referred group and 109 per 1000 in the referred group (RR 2.4). In the group of non-referred, *unexplained asphyxia* still was the most common cause of death, while deaths from *obstetric complications* and *maternal disease* were largely reduced. High relative risks for referred vs. non-referred group were observed for *obstetric complications* (38/1000 vs. 2.1/1000, RR= 18.1), *placental abruption* (RR 8/1000 vs. 0.8/1000, RR=10.0), and *maternal disease* (21.8/1000 vs. 5.5/1000, RR= 4.0).

## Discussion

In this study of perinatal deaths at a zonal hospital in Northern Tanzania during 2000–2010, overall perinatal mortality was 57.7 per 1000 and with no time trends. Among 13 hierarchical categories of perinatal death, major causes were *unexplained asphyxia*, *unexplained stillbirth*, *obstetric complications*, and *maternal disease*. Nearly 20% of the mothers in our study were referred to the hospital for medical reasons, and perinatal mortality in this group was 109 per 1000. Still, perinatal mortality was as high as 45.6 per 1000 in the non-referred group. Mode of delivery of the perinatal deaths corresponded with the figures for all births. The observed distribution of causes suggests a high burden of avoidable deaths if adequate resources were available.

Overall mortality is similar to previous reports from community studies in Kilimanjaro region [7,8], but higher than the National estimates (36-42/1000 births) [3,17]. Mortality is higher than reported in previous studies from the same hospital since both multiple births and births to women referred to the hospital for medical reasons are included in our study [6].

### Maternal disease and abruption placenta

Nearly ninety percent of the deaths in the *maternal disease* category were affected by preeclampsia/eclampsia. Inadequate screening and women’s unawareness of danger signs in pregnancy means that serious maternal complications may go unrecognized leading to a delay in seeking and receiving necessary delivery care. In the Tanzanian demographic and health survey 2004–05, only 47% of the women who attended antenatal clinics recalled having been informed about any danger sign during pregnancy [18]. In a recent study in Rufiji district in Tanzania, 42% of the women who attended antenatal clinics were not informed of any danger signs, whereas only 8.7% were informed of all seven danger signs (vaginal bleeding, severe headache/blurred vision, severe abdominal pain, swollen hands and face, fever, baby stopped or reduced movement, and excessive tiredness or breathlessness) [24]. The risk of perinatal death due to *maternal disease* was in particular high in the referred group (21.8/1000 vs. 5.5/1000, RR=4.0). Also deaths due to *placental abruption*, which is associated with hypertension in pregnancy [25] were more frequent in the referred group (8.0/1000 vs. 0.8/1000, RR=10.0). Screening as well as raised awareness of signs of hypertensive disease in pregnancy among the women and their families could improve pregnancy outcome in this study setting. A study in rural Tanzania indicated that only 9% of all women screened for hypertension by health workers were informed about their blood pressure results [26]. Furthermore, health workers were able to detect high blood pressure level only in four out of the 12 patients with elevated blood pressure levels, of which only one received appropriate counseling. Lack of providers’ ability to screen, counsel and inform women on danger signs seen in rural Tanzania may be present on our area, too. Other threats to improved care are lack of transport, financial constraints, poor compliance with referral, and lack of birth preparedness [27,28].

### Unexplained antepartum stillbirths

*Unexplained antepartum stillbirths* accounted for around half of all antepartum stillbirths. These are deaths largely linked to maternal health conditions before pregnancy, complications of pregnancy, such as preeclampsia, and placental dysfunction, without being able to establish the cause [1]. Antepartum stillbirths are also largely linked to maternal infection and fetal growth restriction [29]. Maternal and fetal infections are estimated to cause about 10-25% of stillbirths in high income countries, whereas, the rate is expected to be higher in low income countries [30]. Studies in low income countries found maternal infections such as syphilis, malaria and HIV are associated with high risk for perinatal morbidity and mortality [31-34]; but infection is not included among the causes of death in the NICE classification. Furthermore, the available information may not be detailed enough to identify the underlying causes for the large fraction of unexplained deaths [13].

### Obstetric causes

In consistence with previous studies from Tanzania [5,7] and other African countries [34-36], obstructed/prolonged labour was present in a majority of the deaths due to *obstetric complications* (80%). Worldwide, obstructed/prolonged labour is the most frequent obstetric complication leading to perinatal mortality [12]. A study in Western Tanzania found obstructed/prolonged labour accounted for 18.5% of perinatal deaths [5]. The risk of perinatal death associated with obstructed labour was 2-fold in Uganda [35], and 8-fold in Kenya [36]. *Obstetric complication* was the category with the largest relative difference between births to non-referred vs. referred women (38.0/1000 vs. 2.1/1000, RR=18.1). It is likely that delay in referral, transportation problems, and delay in taking action for emergency care are responsible for a large fraction of these deaths in the referred group [5,9].

### Unexplained asphyxia

*Unexplained asphyxia* includes intrapartum related stillbirths and intrapartum related neonatal deaths, and was the largest category among births to non-referred mothers. These are deaths closely linked to obstetric and maternal complications and services given during labour and delivery [11,12]; hence they are largely avoidable by appropriate care during time of labour and delivery. The majority of intrapartum stillbirths and early neonatal deaths with Apgar score below 7 at 5 minutes were babies of normal birth weight. These are viable fetuses and babies who would have survived with proper care during labour and delivery. We have previously shown that birth asphyxia was the leading cause of deaths in a neonatal care unit, and that most babies who died due to birth asphyxia had normal birth weight and were born at term [20]. Among interventions recommended to reduce intrapartum stillbirths and deaths related to asphyxia are; appropriate care at birth, the use of partograph for monitoring labour and proper resuscitation for asphyxiated newborns [37]. A qualitative audit study in Tanzania found lack of appropriate fetal heart monitoring during labour in more than 40% of the perinatal deaths [9].

### Strengths and limitations

A major strength of the study is that data is retrieved from a birth registry where the information is collected on a daily basis including holidays with use of a standardized questionnaire. The birth registry is linked to a neonatal registry at the neonatal intensive care unit at the same hospital, where causes of death are routinely registered in a specially designed form. By means of the NICE classification we were able to identify cause of death in more than 50% of the stillbirths. Furthermore, we analyzed a large number of births over a period of 11 years, reducing the possibility of chance findings.

Since the study is based on births in a tertiary care hospital, the results may not be representative for the population because some women are selected to give birth at this level of care due to complications. This might affect overall perinatal mortality as well as the distribution of causes of death. Another limitation is that perinatal mortality may be underestimated since deaths of newborn who were discharged from hospital before seven days were lost if the death occurred at home or in another health facility. Although the standardized birth registry form is designed to collect detailed information, maternal conditions may be underreported due to poor diagnostic procedures in pregnancy, and obstetric conditions may be underreported especially in emergency situations.

## Conclusion and recommendations

Our results indicate that even for deliveries in a tertiary care hospital where emergency obstetric care and neonatal care is available to a larger extent than at lower care levels, there is a huge room for improvement in perinatal outcomes.

First, the high number of deaths in the category of unexplained asphyxia, which also applies to births to women who were not referred for medical reasons, may indicate inadequate monitoring of labour, or inadequate skills on newborn resuscitation immediately after birth. At the hospital level, retraining through ongoing continuous medical training programmes in use of partograph during labour, how to interpret abnormal progress of labour, and basic resuscitation skills might reduce the number of these deaths. Furthermore, feedback mechanisms and regular reviews involving both the obstetric and the neonatal staff should be encouraged. Second, the high number of deaths related to obstetric complications, in particular in the group of referred women, may indicate delay in referral, insufficient referral mechanisms, or delay in seeking health care before labour. Reorganization of the referral system to ensure timely and proper referral, and promoting women and their families to seek health care in time might reduce the number of these deaths. Further studies are needed to identify the most important sources of delay in the referral system, as to be able to implement preventive measures and feedback mechanisms across the referral levels. Third, the high number of deaths related to preeclampsia/eclampsia may indicate unawareness of danger signs related to hypertensive disorders during pregnancy. Both proper screening and counseling on danger signs in connection with antenatal care are relevant measures which may reduce deaths related to maternal conditions.

In conclusion, a future decline in perinatal mortality depends on interventions at different levels. Recommended interventions need to be implemented at the hospital level, with respect to the referral system, and with respect to antenatal care and community education so as to improve perinatal outcomes as soon as possible. The causes of perinatal death identified through the birth registry and the neonatal registry represent valuable information which should be systematically utilized in order to monitor causes of perinatal mortality.

## Competing interests

The authors declare that they have no competing interests.

## Authors’ contributions

BTM: Study design, methodology, data analysis and manuscript writing. RTL, AKD: Study design, methodology, manuscript writing. RO, MJM, OO: manuscript writing. All authors approved the final manuscript.

## Pre-publication history

The pre-publication history for this paper can be accessed here:

http://www.biomedcentral.com/1471-2393/12/139/prepub
